# The Use of Sensors to Prevent, Predict Transition to Chronic and Personalize Treatment of Low Back Pain: A Systematic Review

**DOI:** 10.3390/s23187695

**Published:** 2023-09-06

**Authors:** Pablo Herrero, Izarbe Ríos-Asín, Diego Lapuente-Hernández, Luis Pérez, Sandra Calvo, Marina Gil-Calvo

**Affiliations:** 1 IIS Aragon—iHealthy Research Group, C. de San Juan Bosco, 13, 50009 Zaragoza, Spain; pherrero@unizar.es (P.H.); d.lapuente@unizar.es (D.L.-H.); 735919@unizar.es (L.P.); magic@unileon.es (M.G.-C.); 2 Department of Physiatry and Nursing, Faculty of Health Sciences, University of Zaragoza, C. de Domingo Miral, S/N, 50009 Zaragoza, Spain; iriosasin@correo.ugr.es; 3 Department of Physiotherapy, Faculty of Health Sciences, University of Granada, Av. de la Ilustración, 60, 18071 Granada, Spain; 4 Faculty of Physical Activity and Sports Sciences, Universidad de León, Cjón. Campus Vegazana, S/N, 24007 León, Spain

**Keywords:** biomedical technology, low back pain, outcome assessment, sensor, systematic review

## Abstract

Non-specific low back pain (NSLBP) is a highly prevalent condition that implies substantial expenses and affects quality of life in terms of occupational and recreational activities, physical and psychological health, and general well-being. The diagnosis and treatment are challenging processes due to the unknown underlying causes of the condition. Recently, sensors have been included in clinical practice to implement its management. In this review, we furthered knowledge about the potential benefits of sensors such as force platforms, video systems, electromyography, or inertial measure systems in the assessment process of NSLBP. We concluded that sensors could identify specific characteristics of this population like impaired range of movement, decreased stability, or disturbed back muscular activation. Sensors could provide sufferers with earlier diagnosis, prevention strategies to avoid chronic transition, and more efficient treatment approaches. Nevertheless, the review has limitations that need to be considered in the interpretation of results.

## 1. Introduction

Low back pain (LBP) is considered as one of the most common causes of disability in adults, involving high costs for society [1]. It is estimated that between 5.0% and 10.0% of cases will suffer from chronic LBP at some point in their lives [2]. Global disease research has shown that back pain has a major impact on indirect costs, which can account for between 50% and 89% of the total cost [3]. It is the leading cause of disability, costing more than USD 100 million annually in the USA [3].

Non-specific LBP (NSLBP) is defined as LBP in which it is not possible to detect a specific cause. Patients diagnosed with NSLBP represent 90–95% of the cases of LBP [4,5]. It is known that NSLBP may result from the interaction of different mechanical, anatomical, and psychosocial factors, amongst others, which makes NSLBP assessment challenging for clinicians [4,5]. Chronic back pain diagnosis involves the understanding of the complex interaction amongst the different factors involved in LBP. People with NSLBP are not a homogeneous group, and therefore, the identification of sub-groups of patients with NSLBP who share some characteristics remains a challenge for clinicians in order to provide the best treatment and reduce the burden of the condition. Because of it, an appropriate assessment is essential for providing effective treatment of NSLBP and preventing the transition to a chronic condition, as well as monitoring the patient’s response to treatment in a personalized way [6,7].

Although the most frequent assessments in clinical practice for patients with LBP are made through self-reported measures, sensors have been progressively incorporated in clinical practice to improve the assessment process [8]. The inclusion of sensors in NSLBP diagnosis aims to complement the aforementioned self-reported measures with reliable, objective, and multi-dimensional parameters such as kinematic, kinetic, or electromyographic outcomes. Additionally, sensors could potentially identify common features inside the NSLBP population, which could benefit clinicians by grouping patients with similar patterns, which is a necessary challenge for clinical practice, as discussed above. Finally, sensors could be used by patients to monitor themselves and control their progress in a multi-dimensional way, identifying changes in parameters possibly induced by the pain experience itself or treatment approaches. Nevertheless, their applicability in obtaining valid information needs to be further explored before incorporating these data into the clinical decision-making process. In connection with the latter, the current literature has suggested numerous types of sensors and parameters that may serve to identify NSLBP and it is unknown which ones are more useful in clinical practice [9].

Recently, some reviews have been performed to analyze the use of sensors in patients with LBP from different approaches, such as (i) the analysis of the relationship between the sitting time and the immediate increase in perceived LBP [10], (ii) the applicability for assessing spine movement [9], (iii) the comparison of lumbo-pelvic movement differences [11], (iv) the use of sensors to monitor worker’s activities and to assess biomechanical risk [12], or (v) assessing the contribution of the hip joint and the lumbar segments to assist in determining subgroups within the LBP population [13]. However, there are no reviews that have analyzed the use of sensors in the specific context of personalized medicine for NSLBP.

Therefore, the objectives of this review are (1) to analyze how sensors are used to assess different outcomes related to NSLBP in clinical studies related to prevention, prediction of transition to a chronic condition, or personalization/effectiveness of treatment, (2) to determine if there are sub-groups inside the NSLBP population who have specific characteristics that can be identified with sensors and that require specific treatment strategies.

## 2. Materials and Methods

This systematic review was performed according to the Preferred Reporting Items for Systematic Reviews and Meta-Analyses (PRISMA) standard protocol [14] and has been registered in the International Prospective Register of Systematic Reviews (PROSPERO) (reference number: CRD42022301566).

### 2.1. Eligibility Criteria

Study eligibility and selection was based on the PRISMA checklist PICOS (P—Participants; I—Interventions; C—Comparators; O—Outcomes; S—Study design).

Studies were included in accordance with the following criteria: (1) patients over 18 years of age, including at least a group with chronic (>3 months/12 weeks) NSLBP, with no restrictions regarding race or gender; (2) studies using a sensor system to assess at least one of the following outcomes: lumbar kinematics, lumbar kinetics, and/or electromyography (EMG); (3) studies comparing the chronic NSLBP population with the healthy population, different groups of patients with NSLBP, or the same group pre-post-treatment, or studies exploring the association between outcomes and psychosocial measurements; (4) all types of clinical trials (randomized controlled trials (RCTs), matched controls, cohorts), only considering peer-reviewed journal papers to ensure the credibility of the sources; and (5) publications written in English or Spanish, from 2010 until the present day.

Studies were excluded if they met the following criteria: (1) nonhuman research or trials on animals; (2) publications including people aged under 18 years old; (3) patients who have a specific origin of LBP or have undergone a spinal surgery; (4) publications not specific for the NSLBP population or studying the reliability or validity of different kinds of sensors; or (5) other types of publications such as book sections, conference abstracts or generics, protocols, reviews, or meta-analysis.

### 2.2. Information Sources and Search Strategy

A search of PubMed, MEDLINE, Scopus, and Web of Science (WOS) databases was conducted to collect all available evidence from 2010 to 5 February 2022. Concerning search terms, 3 categories were defined. The first one was related to the population (“low back pain” and “LBP”), the second one was related to the sensor system used (“sensor”, “sensors”, “sensing”, “inertial”, “IMU”, “IMUs”, “accelerometer”, “gyroscope”, “goniom*”, and “body sensor network”), and the third one was related with the outcome measures (“kinematic*”, “kinetic*”, “movement”, “physical activity”, “sedentary behaviour”, “electromyography”, and “EMG”). The choice of these search terms was established after a preliminary literature search and keyword identification. The full search strategies were developed concerning the database in which they were used and the filters applied in Scheme S1 (Supplementary Materials). Furthermore, reference searching was performed to identify additional studies that the database search might have missed.

### 2.3. Study Selection

To decide if the studies met the inclusion criteria, two reviewers screened each report (D.L. and I.R.). They worked independently to avoid bias, following the same methodology after an agreement on the realization of search equations, and then compared their findings. In case of disagreement, a third researcher intervened to reach consensus (M.G.).

Firstly, all the registers were retrieved from the four databases and were introduced in the bibliographic gestor “Mendeley version 1.19.8” in order to remove duplicate publications. After that, a first screening of articles was performed and the articles that could comply with the inclusion criteria regarding the information available in the title and abstract were selected. The process progressed to a second screening phase, in which the studies that passed the previous phase were read in full, and those that fulfilled all the inclusion criteria were selected.

### 2.4. Data Extraction Process

To collect data from studies, the two reviewers worked independently (D.L. and I.R.), and afterwards the reviewers compared the extracted data for consistency. Both reviewers were required to achieve a consensus. All inconsistencies were resolved by discussion between the two data extractors. Any disagreement between the data extractors was solved by involving a third person (M.G.).

### 2.5. Data Items

The following data were extracted from each study: authors, study design, participants’ characteristics (size sample, sex, age, diagnosis, pain duration), intervention (intervention or assessment), comparator, outcomes, and main results.

Regarding outcomes, they were classified in different categories: kinetics (forces, motor control, pressures), kinematics (range of movement, distances, spatiotemporal parameters, accelerometry, angular and linear velocities), EMG (neuromuscular activity), and self-reported measures (related to pain, function, disability, and/or psychosocial factors).

### 2.6. Assessment of Methodological Quality and Risk of Bias

Three different tools were used to assess the risk of bias of the articles included in the systematic review depending on the type of article. The two reviewers worked independently (D.L. and I.R.) and afterwards the results were compared. No intervention from a third reviewer was needed.

For RCTs and studies with random assignment, the “Cochrane Risk of Bias 2.0” (RoB2) tool was used [15]. The assessment of risk of bias was based on ‘assignment to intervention’ for all five domains: (1) randomization process, (2) deviations from intended interventions, (3) missing outcome data, (4) outcome measurement, and (5) selection of the reported result. An overall risk of bias judgement was made for each outcome and each time point as either ‘low risk’, ‘some concerns’, or ‘high risk’ of bias.

On the other hand, for studies with non-randomized interventions, the ROBINS-I tool was used, that is, “Risk Of Bias In Non-randomised Studies—of Interventions” [16], which assesses the risk of bias through seven different domains: (1) confusion, (2) selection of study participants, (3) classification of interventions, (4) deviations from previously stipulated interventions, (5) lack of data or information, (6) measurement of the outcomes, and (7) selection of the exposed results. Each domain was scored based on responses to questions such as ‘low risk’, ‘moderate risk’, ‘high risk’, ‘critical risk’, or ‘no information’. Once the score for each domain was evaluated, the total score for the study was obtained.

Finally, for cross-sectional, case–control, and case-series studies, the National Heart, Lung, and Blood Institute (NHLBI) tools were used: “Quality Assessment Tool for Observational Cohorts and Cross-Sectional Studies”, “Assessment of the quality of case and control studies”, and “Tool for assessing the quality of Case Series studies” [17], respectively. The quality of the studies was assessed by answering a series of questions about the study and the quality of the articles was scored as “good”, “favourable”, or “poor”.

## 3. Results

### 3.1. Study Selection

**Figure 1:**
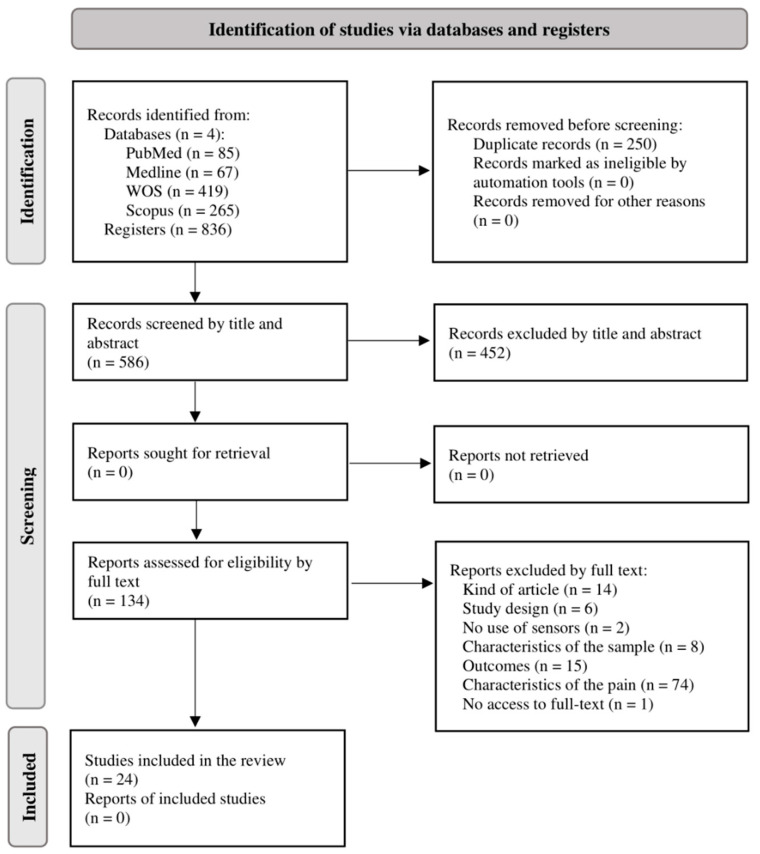
PRISMA flow diagram from search strategy.

As a result of the search through the search strategies described and the filters mentioned, a total of 836 publications were found (PubMed: 85; Medline: 67; WOS: 419; Scopus: 265). After removing duplicate studies, 586 were screened by title and abstract. No additional records were added through other information sources. A total of 134 publications progressed to the second screening phase for a full-text reading, and finally, 24 articles were included in the systematic review for qualitative analysis. The flow diagram (Figure 1) shows in more detail the process of search and selection of studies, together with the different reasons for exclusion, following the PRISMA criteria.

### 3.2. Study Characteristics

The data extracted from the 24 articles are presented in Table 1, grouped according to the sensor system they used to measure the outcomes of interest.

In the 24 articles included, 929 participants reported chronic NSLBP. The average percentage of men forming the samples of chronic NSLBP was 49.32%, while for the women it was 50.68%, with an average age of 38 years old, an average weight of 77.29 kg, an average height of 1.72 m, and a Body Mass Index (BMI) of 24.72 kg/m^2^. The average duration of the pain was reported to be 68.15 months. Concerning the healthy population (502 people), an average of 50.82% men and 49.18% women formed the samples in articles, with an average age of 39 years old, an average weight of 69.60 kg, an average height of 1.72 m, and a BMI of 23.92 kg/m^2^. Apart from the publications concerning the comparison between chronic NSLBP and the healthy population (75%) [18,19,20,21,22,23,24,25,26,27,28,29,30,31,32,33,34,35], 33.33% of articles compared outcomes between different NSLBP groups [20,24,29,36,37,38,39,40] and 25% contrasted outcomes before and after a treatment [24,34,36,37,38,40]. Lastly, three articles (12.5%) analyzed the relationship between outcomes and psychosocial factors [18,25,41]. Only one of them did not compare outcomes by groups or time [41].

As it was, the publications had to use a sensor system to measure at least one of the outcomes. Ten articles (41.67%) used superficial EMG [20,30,31,32,33,34,35,36,37,38], while seven (29.17%) employed inertial sensors [21,22,23,24,39,40,41]. A smaller percentage of articles used photogrammetry systems (20.83%) [20,27,28,29,38], a force platform (20.83%) [28,29,30,31,32] or angular sensors (8.33%) [25,26], among others. Regarding measures, the kinematic outcomes more commonly assessed in the publications were the range of movement (33.33%) [21,22,23,24,25,37,40,41], the spinal axis/angles (33.33%) [20,21,23,24,26,27,28,38], the velocity of movement (20.83%) [25,29,39,40,41], and the spatiotemporal parameters of gait or stride (12.50%) [19,22,30]. Concerning kinetics, measures of the center of pressure (12.50%) were the most repeated [29,31,32]. Finally, regarding EMG measures, muscular activity (41.67%) [20,30,31,32,33,34,35,36,37,38] and strength (20.83%) [19,20,32,33,37] were recurrently measured.

### 3.3. Synthesis of Results

#### 3.3.1. Studies Comparing Population with Chronic NSLBP and Healthy Population

The findings of the different articles selected have made it possible to obtain different results in relation to the outcomes studied. In the case of kinematic measures, most articles found significant differences between the population with and without chronic NSLBP (decreased range of motion, speed, and acceleration, especially in the lumbar flexo-extension movements) [21,22,25,27]. However, a certain discrepancy has been observed in the angles of the spine in the sagittal plane, since two articles did not find significant differences [21,26], while two others did observe significant differences [20,23]. One of these observed that the chronic NSLBP group and healthy group had visual differences in the kyphosis–lordosis axis and significant differences in excursion angle [20]. The other one showed that the chronic NSLBP group, in comparison with the healthy group, had greater error magnitude and variability in the sagittal plane in repositioning the spine in neutral position [23]. In spatiotemporal gait parameters, some differences were found between both groups (shorter step duration, reduced gait cadence and speed [19], higher base of support [30]), but most of them were not significant.

In the case of kinetic measures, the selected studies found that the population with chronic NSLBP presents, with respect to the healthy population, (1) a decrease in gait stability [18]; (2) an increased risk of falls and instability in functional tests [28]; (3) an increase in displacement of the center of pressure when the surface was unstable [31]; and (4) significant differences in postural sway while standing, being greater in the group with chronic NSLBP [31,32]. Regarding the proprioceptive strategy, a single article states that patients with chronic NSLBP depend more on the ankle strategy [31].

The EMG measures indicate that the population with chronic NSLBP has greater muscle activation in the posterior muscles (lumbar spinal erector and lumbar multifidus) in different tasks: walking [30], prolonged standing [20,32,33,35], and sitting [20]. However, no consensus has been reached for the anterior muscles, even though most of the articles show significant differences [20,30,32,34,35]. Results also indicate that populations with chronic NSLBP have less isometric force production in the lower trunk [34] and take longer to reach peak muscle contraction [35].

#### 3.3.2. Studies Comparing Outcomes between Groups of NSLBP

The articles that compared different population groups with NSLBP by active extension/flexion patterns or by chronicity found statistically significant differences between groups that are not evident if they are not sub-classified. For example, in one of them, the velocity of the center of pressure and the total mean velocity were significantly lower in the sub-groups resulting from the “O’Sullivan Classification System” with respect to the healthy population [29]. In another study, the sub-groups resulting from the same classification system had a worse estimate of the neutral position of the spine compared to the healthy population [20]. Moreover, one article differentiated chronic and acute NSLBP, finding that the only variation was that the pain was more intense in those with chronic NSLBP in a weight-lifting test and that the decrease in pain was associated with kinematic changes only in the population with acute NSLBP [40]. Finally, one article used the “STarT Back Assessment Tool” to form groups according to risk of NSLBP progression depending on the movement of the trunk in different directions and planes, in which they found differences in linear acceleration in extension movement, and it was suggested that an extension of 15º in right rotation could be a useful position to identify high-risk NSLBP patients [39].

#### 3.3.3. Studies Comparing Outcomes before and after Treatment

The studies which performed an intervention obtained positive results for the population with chronic NSLBP, improving their disability [37,38], pain intensity [36,37,38,40], postural control [24,36,38], and kinematics [40]. In addition, better results were obtained with specific exercise treatments than with general protocols in two of the studies [36,38], achieving improvements in pain, disability, and in the sense of spinal repositioning.

### 3.4. Assessment of Methodological Quality and Risk of Bias

**Figure 2:**
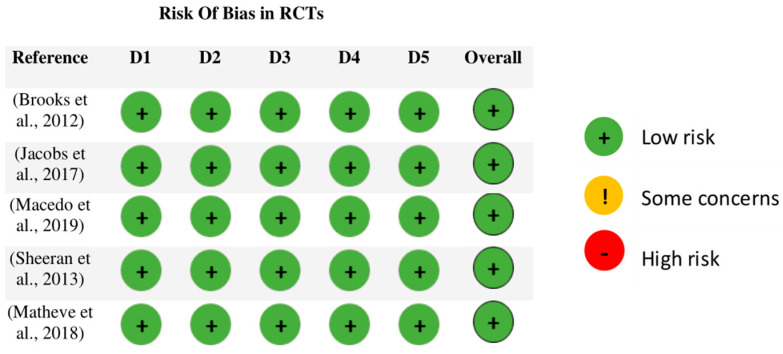
Risk of bias in RTCs with RoB2. Abbreviations: D1, bias due to randomization process; D2, bias due to deviations from intended interventions; D3, bias due to missing data; D4, bias in measurement of outcomes; D5, bias in selection of the reported result. The studies shown in the figure correspond to the following references in the reference list: [24,34,36,37,38].

A total of five RCTs were evaluated with the RoB2 tool. The five articles [24,34,36,37,38] evaluated obtained a “low risk” of bias, in the five domains and in the total (Figure 2).

**Figure 3:**
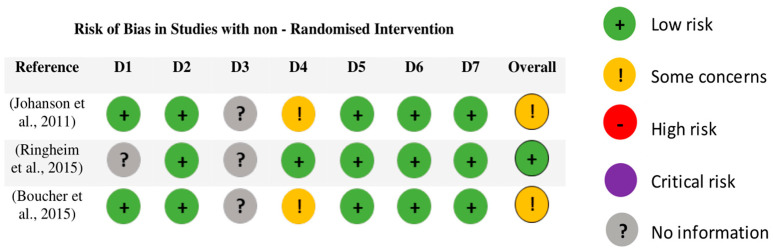
Risk of Bias in Studies with non—Randomized Intervention with ROBINS-I. Abbreviations: D1, bias due to confounding; D2, bias in selection of participants into the study; D3, bias in classification of interventions; D4, bias due to deviations from intended interventions; D5, bias due to missing data; D6, bias in measurement of outcomes; D7, bias in selection of the reported result. The studies shown in the figure correspond to the following references in the reference list: [31,32,33].

The risk of bias of three studies [31,32,33] with non-randomized interventions was also evaluated with the ROBINS-I tool. Of the three studies evaluated, two were found to have “some concerns” in the risk of bias and one obtained a “low risk” of bias (Figure 3).

**Figure 4:**
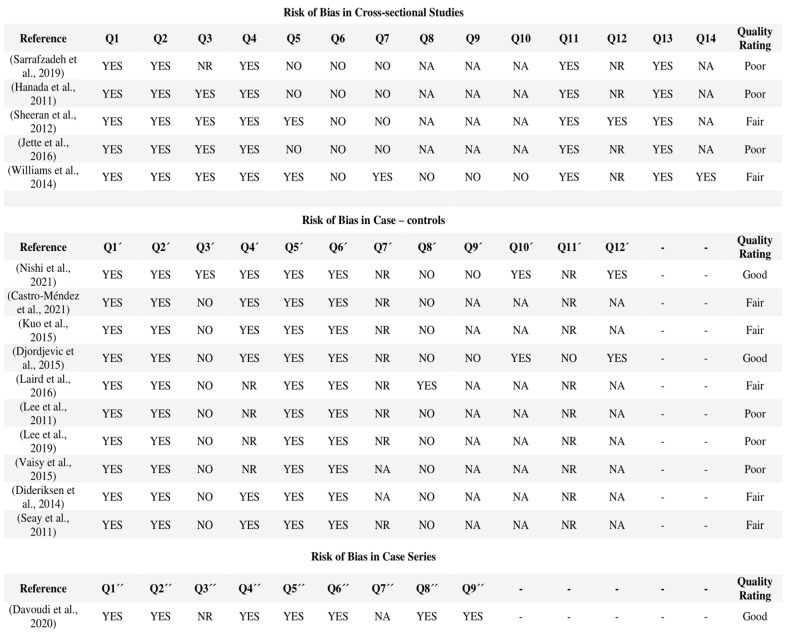
Risk of bias in cross-sectional studies, case–control and case-series with NHLBI. Abbreviations: Q, question; NA, not applicable; NR, not reported. The studies shown in the figure correspond to the following references in the reference list: [18,19,20,21,22,23,25,26,27,28,29,30,35,39,40,41].

Finally, using the NHLBI tools, the quality of five cross-sectional studies [18,20,29,31,40], ten case–control studies [18,19,21,22,23,25,26,27,28,35], and one case-series study [37] were assessed. Of the sixteen studies evaluated, only three were found to have a “good” quality score [18,35,37], seven publications were “favourable” [19,20,21,26,27,28,40], and six were “poor” [22,23,25,29,30,41] in quality (Figure 4).

## 4. Discussion

The main aim of this review was to analyze the use of sensor systems in the assessment of the population with chronic NSLBP in the context of personalized medicine. The results suggest that sensor systems could be effective in identifying some characteristics that could be indicative of chronic NSLBP, playing an important role in the diagnosis, prevention, and progression of this condition. However, there are few outcomes in which the different studies reach a consensus, and the articles related to each other are also very heterogeneous.

With reference to the range of motion, angular velocity, and linear acceleration, statistically significant differences in at least one of these measures have been found between the groups in five publications [21,22,25,27,39], especially in the lumbar flexo-extension movement, with greater differences in extension according to two of them [25,39]. On the other hand, Papi et al. [42] in their systematic review did not reach firm conclusions as to which kinematic and kinetic outcomes could be used to assess people with chronic NSLBP and differentiate them from the rest of the population. In their review, they found some discrepancies regarding differences in range of motion, suggesting that asymmetry of motion, angular velocity, and acceleration might be more effective in assessing people with chronic NSLBP. Regarding spinal angles, the studies reviewed did not agree on the main differences between people with and without chronic NSLBP, finding some studies that showed differences between populations [20,23], while others did not [21,26]. According to Koch and Hänsel [43], there was moderate evidence that spinal angles do not differ between subjects with and without NSLBP, although they did not find high-quality studies. One possible reason for this discrepancy could be, as pointed out by Laird et al. [21], the variability of the anatomy of the hip, pelvis, and spine between individuals.

Differences were observed between the population with and without chronic NSLBP in spatiotemporal parameters in the three articles that analyzed them [18,19,30]. However, only some outcomes were statistically significant, such as length of stride, step, and gait cycle duration. Smith et al. [44] carried out a systematic review on the characteristics of gait in the population with LBP, including all types of temporality and causality, finding that individuals with chronic LBP used reduced gait speed and stride length compared to healthy individuals and that the reduction in kinematic and kinetic parameters could be part of a strategy to reduce the demands of walking in patients with LBP. The coordination of gait phases has also been altered in several studies included in the current review, suggesting that people with chronic NSLBP have a less predictable and more variable gait [18,19]. These findings agreed with the results of Smith et al., who suggest that the population with NSLBP has greater variability of movement patterns in the same phase and relate it to a dysfunction in the dissociation of movement between the trunk and pelvis [44].

A single article stated that patients with chronic NSLBP depend more on the ankle strategy than the healthy population [31]. These results are consistent with those of Claeys et al. [45], who suggested that individuals with NSLBP depended more on the proprioceptive strategy of the ankle and less on that of the hip and spine. It suggested a reduced capacity to switch to a more multi-segmental postural control strategy and less ability to rely on back muscle proprioceptive inputs during complex postural conditions, which leads to decreased postural robustness [45]. However, another study included in the review found that anticipatory postural adjustments did not present significant differences between groups with and without chronic NSLBP, with muscle activation time being similar in both groups [36].

Four of the studies analyzed it either directly or indirectly [18,28,31,32], and their results suggest that the population with NSLBP could have less stability in walking and standing and greater risk of falling than the healthy population.

Regarding EMG measures, most of the articles showed significant differences between the population with and without chronic NSLBP, suggesting that EMG could be an effective method in the early detection of NSLBP and could play an important role in the choice of treatment. Several articles stated that the chronic NSLBP group had higher muscle activation in the posterior muscles (lumbar multifidus and erector spinae) than the healthy population [20,30,32,33,35]. Other articles also found significant differences in the activity of the anterior muscles, but the results were contradictory, with some studies showing more muscle activation in the rectus abdominis [32], external oblique [20,32], and transverse fibers of the internal oblique [20], and others finding less activation in these muscles [30,34]. On the other hand, Ghamkhar and Kahlaee [46] assert that greater muscular activity in the hip and spine in standing may be a strategy to compensate for instability in the spine, which is consistent with the results of this review.

In connection with the latter, a previous review of gait parameters in the LBP population by Smith et al. showed an increased activation of the lumbar paraspinal muscles, which could be related to different phenomena: (1) the reduction in movement and the protection of sensitive and painful tissues; (2) compensation of muscle weakness due to atrophy and fatty infiltration of the multifidus in response to back pain; or (3) proprioceptive alteration [44]. Regarding the abdominal muscles in walking, these same authors concluded that it is highly variable between individuals and that it is more dependent on the speed of locomotion [44]. With regard to muscle strength, most articles measured it in order to determine the % of force used by the different groups in the different functional tasks. One of them compared the maximal voluntary contraction between the groups with and without chronic NSLBP, and it turned out to be lower in the participants with chronic NSLBP in trunk flexo-extension [32]. In addition, one of the articles included in this review showed significant differences in muscle thickness of the transversus abdominis and lumbar multifidus [35], and another ensured that the time to the highest peak of contraction was longer in the population with chronic NSLBP [34].

Two articles related kinematic outcomes with psychosocial parameters. One of them found statistically significant associations between some ranges of movement and “Oswestry Disability Index” scores [41], while another found no significant associations [25]. Another study related psychosocial parameters with impaired trunk control in people with chronic NSLBP [18]. The rest of the studies used different questionnaires and scales but did not analyze their relationship with outcomes measured by the sensors.

Regarding the analysis of the samples by subgroups, two articles [20,29] used the “O’Sullivan classification system” for LBP, which identifies flexor patterns and active extension patterns. In both articles, greater differences were found with the control group when a sub-classification of the population with NSLBP was made. These findings suggest that patients could benefit from more specific, cheaper, and more effective treatments using this rating scale. Papi et al. [42] state that this sub-classification highlights the different pain mechanisms and how they affect function and suggest taking it into consideration in future studies. In another study included in this review [39], the “STarT Back Assessment Tool” was used to classify participants with NSLBP according to the risk of worsening the pathology, observing significant differences in some kinetic outcomes depending on the subgroup. However, none of the articles used sensor systems to classify patients with NSLBP. The absence of studies with sensors on this topic indicates that this is still a topic that requires research in order to determine whether the use of sensors in the diagnosis and assessment of patients could be beneficial as a complement when carrying out assessments, treatments, and/or more specific or personalized follow-up of patients with NSLBP.

In terms of treatment, supervised therapeutic exercise is recommended as the first line of treatment for chronic LBP, despite strong evidence that therapeutic exercise alone is no more effective than conventional physiotherapy methods on chronic LBP [47]. In the current review, two articles [36,38] obtained better results with a specific treatment (therapeutic exercise and postural intervention, respectively) than with the standard care, serving the use of sensors to objectify these improvements. In the European Guideline for the approach of chronic NSLBP, there is limited evidence (Level C) that a home program with specific exercises (individualized, based on a previous evaluation) is more effective than a home program using general exercises, such as those usually included in “back school” activities [47]. In addition, of the six articles that compare outcomes before and after treatment [24,29,34,36,37,38], five of them used physiotherapy treatment (kinesiotaping, postural interventions, therapeutic exercise programs, or feedback with different strategies) [24,34,36,37,38]. Of these, four obtained positive results [24,36,37,38], suggesting that physical therapy could be an effective tool for improving the condition of patients with chronic NSLBP. Likewise, the improvements in the “Oswestry Disability Index” score, pain intensity, postural control, and kinematics suggest that patients with chronic NSLBP are capable of improvement, so each case must be approached individually and personalized to find the most appropriate treatment for the improvement of the patient.

Among the limitations of this review, the heterogeneity of the articles should be highlighted. Publications in which the participants had to perform different functional tasks (functional tests, standing, walking), used different sensor systems, and measured the outcomes with different parameters were included. In addition, to maximize the number of articles, studies that not only compared the population with chronic NSLBP with the healthy population, but also compared the outcomes between groups of NSLBP and assessed the changes before an intervention were included. For this reason, it has been difficult to reach a firm consensus that can be extrapolated and to analyze data quantitatively with a meta-analysis. If there were an adequate number of articles of each type, a more specific review could have been carried out, obtaining more useful and applicable results for clinical practice.

On the other hand, the percentage of articles that obtained little risk of bias in relation to the total is low. According to the “Oxford Table of Evidence Levels” [48], the RCTs constitute level 2 evidence (“1” being the highest level of evidence and “5” the lowest); non-randomized and cross-sectional intervention studies represent level 3; and case–control studies and case-series are included in level 4. This, together with the lack of RCTs that met the inclusion criteria in the review, meant that the risk of bias of the evidence found was high. Likewise, due to the lack of translation resources, the search was limited to articles published in Spanish and English, which could generate a cultural and language bias.

For future research, we suggest performing the following projects: (1) RCTs that use sensors to test their diagnostic capacity; (2) reviews that determine what type of sensors are more effective when assessing a given task in the population with NSLBP; (3) RCTs that assess the efficacy of sensors to classify patients with NSLBP based on one outcome and perform treatments supported by scientific evidence to see if they are more effective than without classifying the population; and (4) studies in which the relationship is investigated between chronic NSLBP and psychosocial parameters such as kinesiophobia, anxiety, or depression, among others. In the same way, it is necessary to establish a protocol to assess these patients which specifies the task or the measurement outcomes that provide the maximum possible information in order to make the best medical decisions. Authors should discuss the results and how they can be interpreted from the perspective of previous studies and of the working hypotheses. The findings and their implications should be discussed in the broadest context possible. Future research directions may also be highlighted.

## 5. Conclusions

In this review, it has not been possible to determine which sensors and outcomes are useful for determining specific characteristics of the population with chronic NSLBP and preventing its appearance and progression or determining the best possible treatment strategy. The heterogeneity of the bibliographic material and the lack of RCTs have made it difficult to obtain consistent results. Even so, findings of interest for clinical practice have been achieved. (1) The population with chronic NSLBP differs from the healthy population in kinematic and kinetic parameters (angular velocity, acceleration, and stability) measured with sensor systems; (2) activation of spinal muscles is greater in the population with chronic NSLBP than in the healthy population, in different functional tasks assessed with EMG; (3) LBP classification systems can form groups that have shown differences in kinetic and kinematic characteristics when they are assessed with sensors, and therefore can benefit from early diagnosis and specific treatments; (4) patients with chronic NSLBP are able to improve with personalized physical therapy treatments; and (5) sensors have the potential to be useful tools in the assessment of NSLBP, but more research is required in this field.

## Figures and Tables

**Table 1:** Characteristics of included studies.

Reference	Study Design	Participants Characteristics	Assessments	Outcome Parameter	Sensor	Scales	Results
[18]	Case-control	LBP group n = 20 Age 54.05 (10.76) Female 9/20 CG n = 20 Age 56.75 (9.43) Female 8/20	Assessment of gait	Accelerations of trunk in A-P (anterior-posterior) and M-L (medium-lateral) MSE (multiscale sample entropy) Stability of gait: LyE (maximum Lyapunov exponent)	Wearable tri-axial accelerometer	NPRS TSK RMDQ	Trunk acceleration ↑ in LBP regardless environment in A-P (* LBP/CG) MSE ↑ in LBP (less predictable and regular patterns) affected by environment in M-L (* LBP/CG) Maximal LyE ↑ in LBP affected by environment in A-P (* LBP/CG), not in M-L (stability ↓ in LBP) Changes in trunk motor control in LBP related to NPRS, TSK, and deficit in ADLs (Activities of Daily Living)
[19]	Case-control, cross-sectional	LBP group n = 75 Female 40.27% CG n = 72 Female 59.72%	Assessment of spatiotemporal gait parameters	Spatiotemporal gait parameters	OptoGait optical sensor system	VAS ODI	Step and gait cycle duration, cadence and velocity ↓ in LBP (* LBP/CG) Step length of both foot ↑ in LBP (* LBP/CG) No association between the laterality of limbs in groups
[20]	Cross-sectional	FP (flexor pattern) n = 51 Age 33.0 (10.3) Female 56.9% AEP (active extensor pattern) n = 39 Age 37.0 (11.4) Female 76.9% CG n = 38 Age 36.0 (10.3) Female 62.9%	Assessment sitting and standing	Spinal repositioning sense: thoracic and lumbar curvatures (AE (absolute error), VE (variable error), CE (constant error)) EMG in LM, ILPT, EO, TrIO, subMVC (Maximal Voluntary contraction)	EMG 3-D kinematic motion analysis system	VAS RMDQ	AE and VE in sitting and standing in thoracic and lumbar spine ↑ in LBP (* LBP/CG) No associations in thoracic and lumbar CE in sitting and standing (* when LBP is classified) TrIO and EO activity in sitting and standing ↑ in LBP (* LBP/CG)/LM and ILPT activity ↑ in LBP (LBP/CG) LM activity in standing ↑ in FP (* FP/CG)
[21]	Case-control	LBP group n = 30 Age 45.8 (11.6) Female 50% CG n = 32 Age 35.5 (12.4) Female 42%	Assessment of lumbar kinematics	ROM, lumbopelvic angles, lumbo-pelvic rhythm (% contribution of the lumbar region to the ROM of the trunk)	ViMove system (Inertial Measurement System)	NPRS RMDQ	No group effect in lordosis angle Pelvic flexion ROM ↑ in LBP; lumbar and thoracic right lateral flexion ↓ in LBP (* LBP/CG) % in flexion ↓ in LBP (more pelvic contribution)
[22]	Case-control	LBP group n = 10 Age 43.2 (12.5) Female 40% CG n = 10 Age 35.9 (16.6) Female 30%	Assessment stair-climbing test, single and double steps	Spatiotemporal stride parameters, ROM in relation to the stride cycle, movement patterns	Spinal motion measurement (3 inertial/magnetic MTx sensors) Stride cycle detection	ODI	No group effect in spatiotemporal parameters Flexion/extension ROM in lumbar spine ↓ in LBP (* LBP/CG) CG: pattern “W” in lumbar sagittal motion (*); LBP: pattern “W” in one test, irregular pattern in other test (single steps) (*)
[23]	Case-control	LBP group n = 17 Age 38.0 (12.0) Female 12/17 CG n = 57 Age 40.1 (16.9) Female 34/57	Assessment during the WPR (Wall Plank-and-Roll) test	General motor patterns (relative movement of thorax and pelvis) Lumbar posture: 3 relative angles (axial twist, kyphosis-lordosis, and lateral bending)	2 inertial sensors	None	Similar patterns LBP/CG, except for a slight displacement ↑ in kyphosis-lordosis angle (visually) and lateral bending at maximal rotation and lateral bending excursion angle ↑ in LBP (* LBP/CG)
[24]	RCT (Randomized Controlled Trial)	LBP group n = 44 (Control n = 15; Mirror n = 15; Sensor n = 14) Age 39.7 Female 18/44 CG n = 47 (Control n = 17; Mirror n = 15; Sensor n = 15) Age 36.7 Female 24/47	Assessment at baseline, during and after intervention (lumbopelvic control task with feedback from sensors, mirror or no feedback)	Lumbopelvic kinematics measurements in the sagittal plane	Laptop 3 wireless inertial measurement sensors (Valedo motion research tool)	NPRS RMDQ TSK	Sensor group ↑ in LBP and CG, for both tasks and in lumbar spine and hip (* control/mirror/sensor) No group effect (control/mirror/sensor) in post-intervention questionnaire LBP equally capable of ↑ lumbopelvic movement control
[25]	Case-control	LBP group n = 20 Age 32.9 (9.6) Female 55% CG n = 19 Age 29.1 (7.1) Female 48%	Assessment of analytic movements in two phases: ascendant and descendent	Maximal ROM, time to maximal ROM, maximal and average angular velocity of spinal movement	Epionics SPINE (two strips with 12 angle sensors per strip)	SF-STAI ODI TSK PCS NPRS SF-36	Maximal ROM ↓ in LBP (in lumbar extension a 35%<)/Time to maximal pelvic and lumbar flexion ↑ in LBP (* LBP/CG) Angular deflection: lumbar and pelvic peak angular deflection ↓ in LBP/Time to maximal deflection and return to baseline ↑ in LBP/Maximal angular velocity in maximal deflection ↓ in LBP/Average angular velocity ↓ in LBP in extension in more subunits (12/18) tan in flexion (8/18) ROM and maximal angular velocity in descending phase of movement no related to PCS, SF-STAI, TSK/Correlation in units 1 and 2 in velocity/No correlation between kinematics and ODI, SF-36, NPRS (*)
[26]	Case-control	LBP group n = 17 Age 32.5 (9.6) Female 59% CG n = 17 Female 53%	Assessment of spine kinematics during a repetitive lifting task	Angles in sagittal plane, variance and offset (average) of spinal angles % of determination of accessory movement	Epionics SPINE	TSK PCS ODI SF-STAI SF-36 NPRS	No group effect in task-related angles Little difference in the magnitude of accessory angles LBP/CG (* at sensor 2) % of determination ↑ in LBP (* LBP/CG), variability of muscular activation pattern is less changing
[27]	Case-control	LBP group n = 14 Age 35.71 (10.90) Female 6/14 Recovered from LBP n = 14 Age 32.56 (9.42) Female 5/14 CG n = 14 Age 29.90 (8.45) Female 8/14	Assessment while walking at systematically increased speed	3-D pelvis and trunk segment angles and coordination between pelvis and trunk (changes in angle-angle diagrams)	8 high-speed cameras	ODI VAS	Time in gait cycle in-phase in the frontal plane ↑ in LBP while walking (* LBP/CG) Pelvis axial rotation and time in gait cycle in-phase in the transverse plane ↑ in LBP while running (* LBP/CG) Antiphase coordination time ↓ in LBP and recovered LBP while running (* LBP/recovered/CG) Pelvis and trunk motion ↑ in LBP in the sagittal plane as walking speed increased (* LBP/recovered/CG)
[28]	Case-control	LBP group n = 13 Age 60.5 (4.1) Female 9/13 CG n = 13 Age 59.7 (3.0)	Assessment during single leg-standing, TUG (Timed Up and Go) and 5TSTS (Five Times to Sit To Stand test)	Steadiness index of spinal regions RHT (relative holding time) and RST (relative standstill time) Time TUG and 5TSTS	6-camera motion analysis system (Vicon) AMTI Force platform	ODI	N° people capable of stay 20 s in single leg-standing ↓ in LBP (* LBP/CG) Time TUG and 5TSTS ↑ in LBP (* LBP/CG) RHT ↓ in LBP (* LBP/CG) RST in non-painful side of trunk, thoracic and lumbar spine ↓ in LBP (* LBP/CG), LBP less stable in single leg-standing RST in painful side ↓ in LBP (LBP/CG) TUG and 5TSTS no correlated with RHT and RST
[29]	Cross-sectional	FP group n = 20 Age 32.42 (8.36) AEP group n = 20 Age 33.05 (9.01) CG n = 20 Age 31.06 (8)	Assessment of lifting test (static and dynamic phase)	Postural balance: SD.apx (SD (standard deviation) of COP amplitude in the frontal plane); SD.Apy (SD of COP amplitude in the sagittal plane); SD.APvx (SD of COP velocity in frontal plane); SD.SPvy (SD of COP velocity in sagittal plane), MTV (mean total velocity)	6-camera motion analysis system Force plate system	VAS	No association LBP/CG in any outcome, except SD.APvx in static phase (↓ in LBP) Dynamic phase: SD.APvx, SD.APvy and MTV ↓ in AEP (* AEP/GC), ↓ in FP (FP/GC) Static phase: SD.APvx, SD.APvy and MTV ↓ in AEP and FP (* AEP, FP/GC) Less postural sway in LBP, classification effect
[30]	Cross-sectional	LBP group n = 9 Age 61.4 (9.8) Female 5/9 CG n = 9 Age 64.9 (8.8) Female 5/9	Assessment during 4 walking trials, at rest and at the MVIC (Maximal Voluntary Isometric Contraction)	EMG in low RA, LES, IO, and LM MVIC Spatiotemporal gait parameters	GAITRite mat with pressure sensors EMG (surface electrodes)	ABCSQ RMDQ	Activity low RA (% of MVIC) ↓ in LBP (* LBP/CG) Activity LES ↑ in LBP in 4 subphases (* LBP/CG) Activity right IO ↓in LBP in Right Mid Stance (phase 4) (* LBP/CG) Activity right LM ↑ in LBP in 4 subphases (* LBP/CG) No association in demographic, functional scales or gait parameters LBP/CG
[31]	Intervention no randomized	LBP n = 16 Age 22.0 (1.1) Female 11/16 CG n = 16 Age 22.7 (1.7) Female 11/16	Assessment of an endurance test, with and without BMF (back muscle fatigue)	Displacement of COP, positions of COP in A-P, RMS (root mean square)-COP, ratio of COP displacement	Force plate EMG mean power frequency	ODI NPRS Borg Scale	Normal conditions: RMS-COP ↑ in LBP in stable support (* stable/unstable)/Posterior sways in vibration TS ↑ in LBP; anterior sways in vibration LM ↓ in LBP (* LBP/CG)/Ankle-steered proprioceptive control strategy ↑ in LBP (* LBP/CG) BMF: Endurance time ↓ in LBP (* LBP/CG) After BMF: postural stability ↓ in CG with unstable support surface, LBP maintained ↓ postural stability (* LBP/CG)/Anterior sway in vibration LM ↓ in LBP (* LBP/CG); BMF no influence on relative proprioceptive weighting ratios in LBP with an unstable support surface (*)
[32]	Intervention no randomized	LBP group n = 17 Age 39.0 (5.4) Female 10/17 CG n = 20 Age 40.2 (5.4) Female 13/20	Assessment of three standing tests: quiet standing, prolonged standing and quiet standing	EMG in ES, GM, RA, EO MVC in flexo-extension Force reaction in quiet standing (global COP, COP RMS, COP speed A-P and M-L, COP area) Number of shifts in body weight	Force sensor Force plates EMG (surface electrodes)	VAS ODI TSK Borg Scale NPRS	Prolonged standing: Body weight shifts and postural sway values in all COP outcomes ↑ in LBP (* LBP/CG in COP speed, a trend for COP area and A-P RMS-COP)/Relative muscle activation level ↑ in LBP for all muscles except GM Quiet standing: No association in groups in COP outcomes (before, after)/Trunk extension-flexion strength ↓ in LBP before and after (* LBP/CG), ↓ in both groups after/before (*)
[33]	Intervention no randomized	LBP group n = 20 Age 33.7 (14.4) Female 7/20 CG n = 20 Age 29.1 (7.8) Female 7/20	Assessment during isometric contractions and during the fatigue protocol	EMG in LES Force data: TPT (time to peak torque), CE, VE, AE	EMG (surface electrodes)	ODI TSK VAS	Activity in LES in L4-L5 in no fatigue ↑ in LBP (* LBP/CG) CE, AE ↑ in LBP in no vibration (* LBP/CG); more ↓ of CE and AE in LBP with vibration (* LBP/CG) (↓ precision in the reproduction of trunk strength) Effect of fatigue: activity in LES in L4-L5 ↓ in LBP (* fatigue/no fatigue)/No group effect in MVC before and after fatigue and in TPT (both ↑ after)
[34]	Planned secondary analysis of a prospectively registered RCT	LBP group n = 68 Age 41.1 (38.8–44.0) Female 44% CG n = 27 Age 32.5 (28.8–36.2) Female 67%	Assessment 1 week before/after treatment (stabilisation protocol and MSI-directed protocol)	EMG in EO, IO, RA Responses to lateral, forward, and backward perturbations	EMG (surface electrodes) Force platform (disturbances)	ODI NPRS	Integrated EMG amplitudes in EO, IO and RA ↓ in LBP (* LBP/CG) No effect of treatment in integrated EMG amplitudes (*)
[35]	Case-control	LBP group n = 36 Age 53.22 (8.12) Female 18/36 CG n = 37 Age 52.55 (9.45) Female 22/37	Assessment at rest and contraction	EMG in TrA, LM Muscle thickness	Wireless LUMBIA System Ultrasound	VAS ODI	TrA thickness at rest ↑ in LBP; TrA thickness in maximal contraction ↓ in LBP; relative change in TrA thickness ↓ in LBP (* LBP/CG) No group effect in LM thickness in rest or contraction; relative change in LM thickness ↓ in LBP (* LBP/CG) Normalized amplitudes of EMG in TrA ↓ in LBP; in LM ↑ in LBP (* LBP/CG)
[36]	RCT	SEG group n = 32 Age 36.2 (8.2) Female 20/32 GEG group n = 32 Female 20/32	Assessment of APAs (Anticipatory postural Adjustments) in rapid shoulder flexion, before and after intervention	APAs EMG in RA, LES, TrA, IO	EMG (surface electrodes)	VAS ODI	ODI ↓ in SEG (* SEG/GEG) VAS ↓ in SEG and GEG (↓ 30% in SEG) (*) APAs were not delayed in LBP, and ↑ after intervention in both groups
[37]	Assessor blinded prospective RCT	Kinesiotaping + tension (KTT) n = 27 Age 25 (6) Kinesiotaping no tension (KTNT) n = 27 Age 24(5) Micropore (MP) n = 27 Age 25 (5) CG n = 27 Age 24 (4)	Assessment pre, 3 and 10 days after intervention (Kinesiotaping or Micropore tape)	Trunk ROM (range of movement) EMG in longissimus muscles MVIC	EMG (surface electrodes) iHandy level (iPhone app, ROM)	NPRS RMDQ	Pain relief ↑ in KTT, KTNT 3 days after application of the tape (* KTT, KTNT/CG) Disability ↓ in KTT at 3 and 10 days (* KTT/CG) No association in all the other outcomes
[38]	Pragmatic RCT single-blinded	CSPI group n = 25 Age 35.9 (10.13) Female 64% GPI group n = 24 Age 37.1 (11.1) Female 54.2%	Assessment sitting and standing Treatment: CSPI/GPI	EMG in LM, ILPT, EO, TrIO Spinal repositioning sense: thoracic and lumbar curvatures (AE, VE, CE)	8-channel EMG (surface electrodes) 3-D kinematic Vicon motion analysis system	VAS RMDQ	Thoracic and lumbar AE in sitting and lumbar AE in standing ↓ in CSPI (* CSPI/GPI/time), same trend in the rest of outcomes Muscular activity ↓ in CSPI (CSPI/GPI) Lumbar CE in standing ↑ in CSPI (* CSPI/GPI/time) No differences after home-based intervention CSPI more effective in reducing pain and disability and in increasing spinal repositioning sense, not effect in muscular activity
[39]	Case series	LR (Low risk) n = 33 Age 46.1 (3.2) MR (Medium risk) n = 35 Age 44.8 (4.4) HR (High risk) n = 32 Age 44.3 (3.4)	Assessment of trunk flexo-extension in 5 planes of movement at maximum speed without pain	Maximal and average angular velocity, linear acceleration and maximal jerk (2nd derivate of the angular velocity)	Inertial sensor	VAS	Maximal and average velocity and maximal and average acceleration ↓ with the increase in movement asymmetry (from 0° to 30° in flexion-extension, in 3 subgroups) Average velocity and maximal jerk ↑ in extension; average acceleration ↑ in flexion (* flexion/extension)/Linear acceleration associated with subgroup (*) Differences between groups more evident in extension: Maximal jerk ↑ in HR and MR (* HR/MR/LR)/Extension in sagittal plane * HR/(MR + LR)/Acceleration in movement planes ↓ in HR (specially at 0°, * HR/MR/LR) Extension of 15° of right rotation could be useful to diagnose LBP of HR
[40]	Prospective, cross-sectional, experimental repeated-measures design	Acute LBP n = 20 Age 42.7 (6.8) Female 9/20 Chronic LBP n = 20 Age 36.6 (10.8) Female 9/20	Assessment before and after intervention (oral analgesia)	Lumbar ROM, angular velocity, angular acceleration	Two wired 3DM-GX3-25 inertial sensors	VAS TSK	No group effect at baseline, except for duration and pain intensity ROM evoked pain ↓ after treatment in both groups (*) Pain ↑ in chronic LBP in lifting (* between groups) No interactions group/time No correlation between ↓ pain and kinematic change in chronic LBP; correlation between ↓ pain and ↑ of velocity in flexion, ROM and acceleration in right lateral bending in acute LBP (*).
[41]	Cross-sectional	n = 32 Age 32.94 (7.83) Female 59.4%	Assessment of lumbar kinematics	Maximal ROM, maximal minimum angular velocity, acceleration	Inertial Measurement System	VAS ODI FABQ	ODI (functional disability) correlated with certain lumbar kinematic parameters: Moderate +: ROM right lateral flexion and rotation; maximal angular velocity lateral flexion L1; minimum angular velocity left lateral flexion S2. Weak-moderate −: ROM left rotation; maximal angular velocity left lateral flexion S2; minimum angular velocity lateral flexion L1, right rotation L1 and left rotation S2. No correlation kinematics—FABQ.

Caption: ABCSQ: Activities-specific Balance Confidence Scale Questionnaire, BMI: body mass index, CG: control group, COP: center of pressures, CSPI: Classification System-guided Postural Intervention, EMG: electromyography, EO: external oblique, FABQ: Fear avoidance beliefs questionnaire, GEG: General Exercise Group, GM: gluteus medius, GPI: General Postural Intervention, ILPT: iliocostalis lumborum pars thoracic, IO: internal oblique, LBP: low back pain, LES: lumbar erector spinae, LM: lumbar multifidus, NPRS: Numeric Pain Rating Scale, NSCLBP: non-specific chronic low back pain, ODI: Oswestry Disability Index, PCS: Pain Catastrophizing Scale, RA: rectus abdominis, RMDQ: Roland–Morris Disability Questionnaire, ROM: range of movement, SEG: Specific Exercise Group, SF-36: Short form—Health Survey, SF-STAI: Spielberger State–Trait Anxiety Inventory, TrA: transverse abdominis, TrIO: transverse fibers of internal oblique, TS: triceps surae, TSK: Tampa Scale of Kinesiophobia, VAS: Visual Analogue Scale. Participants characteristics: data are presented as mean (standard deviation). Results: * significant difference; an increase of the corresponding outcome parameter or scale is represented with ↑; a reduction of the corresponding outcome parameter or scale is represented with ↓.

## Data Availability

The data presented in this study are available on request from the corresponding author.

## Supplementary Materials

The following supporting information can be downloaded at: https://www.mdpi.com/article/10.3390/s23187695/s1, Scheme S1: Search strategies; Assessment tool 1: Cochrane Risk of Bias 2.0 https://www.riskofbias.info/welcome/rob-2-0-tool/current-version-of-rob-2 (accessed on 1 September 2023); Assessment Tool 2: Risk of Bias in Non-Randomised Studies of Interventions https://www.riskofbias.info/welcome/home/current-version-of-robins-i (accessed on 1 September 2023); Assessment Tool 3: Quality Assessment Tool for Observational Cohorts and Cross-Sectional Studies https://www.nhlbi.nih.gov/health-topics/study-quality-assessment-tools (accessed on 1 September 2023); Assessment Tool 4: Assessment of the Quality of Case and Control Studies https://www.nhlbi.nih.gov/health-topics/study-quality-assessment-tools (accessed on 1 September 2023); Assessment Tool 5: Tool for Assessing the Quality of Case Series Studies https://www.nhlbi.nih.gov/health-topics/study-quality-assessment-tools (accessed on 1 September 2023).
