# Supplementary material for: The Use of Sensors to Prevent, Predict Transition to Chronic and Personalize Treatment of Low Back Pain: A Systematic Review
Source: Sensors (Basel). 2023 Sep 6;23(18):7695. doi: 10.3390/s23187695 (PMC10534870; doi:10.3390/s23187695)
Supplement: Supplementary file 1 [file sensors-23-07695-s001.zip › sensors-2529452-supplementary.pdf]

sensors MDPI

Supplementary Materials:

Scheme S1. Search strategies

- PubMed → (("Low back pain" [Mesh] OR LBP) AND (sensor OR sensors OR sensing OR inertial OR IMU OR IMUs OR accelerometer OR gyroscope OR goniom* OR "body sensor network") AND (kinematic* OR kinetic* OR movement OR "physical activity" OR "sedentary behaviour" OR "heart rate" OR electromyography OR EMG))

- Medline → noft("Low Back Pain" OR LBP) AND noft(sensor OR sensors OR sensing OR inertial OR IMU OR IMUs OR accelerometer OR gyroscope OR goniom* OR "body sensor network") AND noft(kinematic* OR kinetic* OR movement OR "physical activity" OR "sedentary behaviour" OR "heart rate" OR electromyography OR EMG) → filter (RCT OR Comparative Study OR Observational Study OR Clinical Trial OR Clinical Trial Protocol OR Controlled Clinical Trial)

- WOS → ((TS=("Low back pain" OR LBP)) AND TS=(sensor OR sensors OR sensing OR inertial OR IMU OR IMUS OR accelerometer OR gyroscope OR goniom* OR "body sensor network") AND TS=(kinematic* OR kinetic* OR movement OR "physical activity" OR "sedentary behaviour" OR "heart rate" OR electromyography OR EMG)) → filter (article)

- Scopus → (TITLE-ABS-KEY ("Low back pain" OR lbp) AND TITLE-ABS-KEY (sensor OR sensors OR sensing OR inertial OR imu OR imus OR accelerometer OR gyroscope OR goniom* OR "body sensor network") AND TITLE-ABS-KEY (kinematic* OR kinetic* OR movement OR "physical activity" OR "sedentary behaviour" OR "heart rate" OR electromyography OR emg)) AND PUBYEAR > 2009 AND (LIMIT-TO (DOCTYPE , "ar")) → filter (articl
